# Use of appropriate healthcare technologies: a cross-sectional study in rural Zhejiang of China

**DOI:** 10.1186/s12913-015-0947-4

**Published:** 2015-07-29

**Authors:** Jianping Ren, Chaojie Liu, Qi-Sheng Gao, Lianping Yang, Xianhong Huang, Qing Guo

**Affiliations:** School of Medicine, Hangzhou Normal University, Hangzhou, Zhejiang 310036 China; School of Management, Hubei University of Chinese Medicine, Wuhan, Hubei 430065 China; School of Psychology and Public Health, La Trobe University, Melbourne, 3086 Australia; Zhejiang Higher Medical College, Hangzhou, Zhejiang 310036 China; School of Medicine and Health Management, Tongji Medical College, Huazhong University of Science and Technology, Wuhan, Hubei 430030 China

**Keywords:** Appropriate healthcare technology, Primary care, China

## Abstract

**Background:**

Appropriate healthcare technologies (AHTs) are an important strategy for improving the availability and accessibility of healthcare services. It is not clear what impact AHTs have on health workers and consumers; and whether those AHTs can continue in place without special or ongoing financial support. This study investigated the attitudes of health workers and consumers towards AHTs.

**Methods:**

Health facilities from five counties in Zhejiang were surveyed. Participants of the study included all health workers who were involved in the delivery of AHTs in the selected organizations and a group of randomly selected patients who sought services from the participating organizations. A total of 822 questionnaires from health workers and 693 questionnaires from patients were collected for data analyses. The questionnaires measured perceptions and attitudes of respondents towards AHTs using a Likert scale.

**Results:**

The respondents delivering public health services rated the highest scores to AHTs (4.42 ± 0.7), followed by those engaged in management of chronic conditions (4.41 ± 0.57) and Traditional Chinese Medicine (TCM) (4.29 ± 0.55). Around 90 % of health workers believed that AHTs were meaningful for rural patients; however, only 69 % of health workers believed that the technologies encouraged by the government were sufficiently developed or “mature”, and more than 24 % acknowledged difficulties in using those technologies. Overall, patients were satisfied with AHTs, with 71.6 % feeling “very satisfied” or “satisfied”, 24.2 % feeling “acceptable” and 1.6 % feeling “dissatisfied”. Most (83 %) patients were satisfied or very satisfied with Traditional Chinese Medicine, compared with management of chronic conditions (80 %), family planning (67 %), public health services (64 %), and finally with maternal and child health care (59 %).

**Conclusions:**

Local acceptability should be taken into consideration in determination of AHTs; consumer health literacy needs improvement, particularly in relation to public health and preventive services.

## Background

Appropriate Healthcare Technology (AHT) refers to “methods, procedures, techniques and equipment that are scientifically valid, adapted to local needs and acceptable to those who use them and to those for whom they are used, and that can be maintained and utilized with resources the community or country can afford” [1]. AHTs have been designated by the World Health Organization (WHO) and recognized by many resource-poor countries as an important improvement strategy for increasing the availability and accessibility of healthcare services [2]. As Lumbiganon [3] puts it: “AHTs have to be effective, safe, affordable and acceptable; and it is particularly important for the resource-poor countries to select a limited range of AHTs to maximize their return on investment”.

In the international literature, AHTs have often been reported as a component within aid programs, exporting essential technologies from developed nations to developing nations [4]. These exported technologies usually involve stringent requirements in terms of their adequacy and the ongoing ability of recipients to implement, manage, and maintain those technologies [5]. Soft technology that brings about changes in behaviors in recipient communities is regarded as equally important, if not more, compared to hard technology or infrastructure: therefore, quality training is highly appreciated. The major disadvantage of exported technology is that they are usually focused on a specific health issue and often lack comprehensiveness. For example, the *Program for Appropriate Technology in Health* (commonly known as PATH), a large international organization based in the US, has played a significant role in defining and promoting AHTs in relation to maternal and child health, and control of infectious diseases [6]. PATH actively engages in research, surveillance, training and promotion activities. In recent years, PATH’s scope of services has extended to the management of chronic conditions [7].

Recently there has been an increasing call for self-reliance regarding the development of AHTs [1]. Unfortunately, research documenting self-determined comprehensive AHT programs is limited. This study was conducted in China, focusing on an AHT initiative that does not involve any export–import relationship and is comprehensive in nature.

Due to the rapid economic development and increasing governmental investment in health over recent decades, China is successfully rejuvenating its health care networks. However, the improved conditions of health facilities (eg. infrastructure and human resource development) have failed to directly translate into improved health outcomes. The burden of disease, especially for the rural residents, remains very high; and the population continues to complain about soaring medical expenses (which often involve excessive and unnecessary services) not covered by health insurances that must be paid out-of-pocket [8]. The *National Health Survey* showed that 30.3 % in 2003 and 24.7 % in 2008 of rural residents disregarded their doctor’s recommendation for admission to hospital, mainly in relation to financial affordability [9, 10]. A lack of standardization, particularly with respect to technology configuration, and the excessive use of inappropriate and expensive technologies related to the pursuance of financial interests by health providers contribute to these problems. Meanwhile, many cost effective and more appropriate services remain unavailable in primary care facilities [11].

The Ministry of Health (MOH) of China introduced AHTs as an effective strategy for improving accessibility and equity of healthcare services in resource-poor settings. Such a vision can be traced back to 1991, when the MOH developed a ten-year plan to promote AHTs in rural China. Although the market-oriented health reform seriously restricted the capacity to encourage health facilities to adopt AHTs, by 2001 the MOH established 130 AHTs in rural health facilities through the training of 250,000 health workers. It is reckoned that this initiative avoided unnecessary medical expenditure, some hundreds of millions of Yuan, that rural residents would have otherwise had to fund [12].

Unfortunately, AHTs are not widely available in all rural health facilities. In 2004, to demonstrate the effectiveness of AHTs, the MOH selected 20 rural health facilities across 10 provinces to implement AHTs more systematically. In 2007, the demonstration project was extended to a further 15 provinces.

It is important to acknowledge that the selection process of AHTs for rural China is characterized by some unique features. First, apart from considerations of cost, safety and effectiveness, a balance between western and traditional Chinese medical technologies is included in the selection of AHTs. Second, despite this being a top-down initiative of the MOH, local governments have autonomy to expand the AHT list. Third, different AHTs apply to the three levels of rural health facilities (county, township and village). Finally, the pilot and demonstration project is accompanied with project funding support from the governments.

It is unclear what impact AHTs have on health workers and consumers; and as a result, whether those AHTs can sustain without special funding support. This study aims to investigate the attitudes of health workers and consumers towards AHTs for this reason.

## Methods

### Study setting and participants

This study was undertaken in Zhejiang province in the eastern developed region of China, covering an area of more than 100,000 km^2^. It has a population of 54.43 million: one of the highest population densities in China. In 2012, the GDP per capita in Zhejiang reached 63346.70 Yuan, ranking in the highest range of all provinces [13].

This study obtained ethics approval from the Ethics Committee of Hangzhou Normal University Hospital, and no personal identification information was included in data analysis.

Five counties in Zhejiang were identified by the MOH as pilot and demonstration sites of its AHT initiative, among which three were deemed as developed countryside (Yuhang, Zhuji, Shaoxing), one as developing (Jiande) and one under-developed (Anjie). We investigated all of the county level health facilities in the five counties, which included hospitals, maternal and child health care centers, centers for disease control and prevention, and family planning institutions. A simple random sampling method was adopted for selecting township level health facilities. Four township health centers were selected in each county.

Participants of the study included all health workers in the selected organizations who were involved in the delivery of AHTs. The participants covered the areas of public health services, medical services, maternal and child care, traditional Chinese medicine (TCM), and family planning services. A total of 1000 questionnaires were distributed to eligible participants, and 886 (88.6 %) questionnaires were returned: of these, 822 (92.8 %) were valid for data analyses.

We also conducted a survey of people who sought services from the participating organizations (patients). Each county was instructed to identify 15 technologies (from the AHT list) and complete ten questionnaires for the patients who received services associated with each of the technologies. Data were collected in January 2010 and 750 questionnaires were distributed. Respondents of the questionnaire were those who were 12 years or older, or the parents of those who were younger than 12. A total of 703 (93.7 %) questionnaires were returned. Of the returned questionnaires, 693 (98.6 %) were valid for data analyses. Due to capacity constraints, patients who did not use the AHTs were not included in the survey.

### Survey instrument

We developed two separate attitudinal response questionnaires using 5 point Likert scales, that asked either health workers or patients to rate their experience with AHTs. The questionnaire for health workers contained: 4 items measuring perceived maturity and difficulty of AHTs; 4 items measuring attitudes towards the impact of AHTs on patients (acceptance, effectiveness and financial burden); 2 items measuring willingness to use AHTs; 4 items measuring the impact of AHTs on health workers themselves (career, technical competency and income); and 6 items measuring satisfactions on training and management of AHTs. The questionnaire for patients measured perceived effectiveness of AHTs (1 item), and satisfaction on medical services (1 item) as well as on technologies (1 item) and on expenses (1 item) involved in the services.

### Data collection

Questionnaires were distributed to eligible health workers by the AHT project leaders within each participating organization. Health workers who agreed to participate in this study completed their own questionnaire first, and were then asked to invite their patients (if eligible) to complete the patient questionnaire. Completion of both questionnaires was voluntary and anonymous.

### Data analysis

All statistical analyses were performed using SPSS16.0.

Each questionnaire item was measured using a five point Likert scale (ranging from 1 “very unsatisfied” to 5 “very satisfied”). A dimensional score was computed by calculating the average score of items in each dimension [14]. The differences of dimensional scores across different areas of services and demographic characteristics of the respondents were tested using Mann–Whitney *U* test or Kruskal-Wallis H test because the scores were not normally distributed [15].

The differences of patient rating on the level of price of AHT services across different areas of services and demographic characteristics of the respondents were tested using Chi-square tests because only one item was included in this dimension.

An aggregate score (the sum of all item scores) was computed for the health worker cohort and separately for the patient cohort. These summed scores served as dependent variables in multiple linear regression analyses for the purpose of identifying independent variables that had a significant association with the aggregate score. A forward stepwise regression based on the maximum likelihood estimation method was adopted, with an enter/exit criterion (α) of 0.05/0.10 [14].

The independent variables entered into the regression analysis for health worker scores comprised: demographic characteristics, educational attainments, income, work experience and expertise (Table 1), while those for patient scores included: demographic characteristics, educational attainments, income, health insurance and technologies used (Table 2).

**Table 1:** Independent variables included in the regression analysis on health worker scores

Independent variable	Coding values
Age (x1)	Continuous
Gender (x2)	Female = 0, Male = 1
Level of Education	
x31	Lower than junior high school = 0, Senior high (vocational) school = 1
x32	Lower than junior high school = 0, Associate degree = 1
x33	Lower than junior high school = 0, Bachelor degree or higher = 1
Monthly Income, Yuan (x4)	Below 1000 = 0, 1000-2999 = 1, 3000-4999 = 2, 5000 and above = 3
Professional Title	
x51	No title = 0, Junior level = 1
x52	No title = 0, Middle level = 1
x53	No title = 0, Senior level = 1
Work Experience, Year (x6)	Below 9 = 0, 10-19 = 1, 20-29 = 2, 30 and above = 3
Area of Technologies	
x71	Maternal and child care = 0, Public health = 1
x72	Maternal and child care = 0, Traditional Chinese medicine = 1
x73	Maternal and child care = 0, Family planning = 1
x74	Maternal and child care = 0, Chronic conditions = 1
Financial Incentives (x8)	No = 0, Yes = 1

**Table 2:** Independent variables included in the regression analysis on patient scores

Independent variable	Coding values
Age (x1)	Continuous
Gender (*x*2)	Female = 0, Male = 1
Level of Education	
x31	Lower than junior high school = 0, Senior high (vocational) school = 1
x32	Lower than junior high school = 0, Associate degree = 1
x33	Lower than junior high school = 0, Bachelor degree or higher = 1
Annual Household Income (x4)	Below 10000 = 0, 10000-29999 = 1, 30000-49999 = 2, 50000 and above = 3
Health Insurance (x5)	No = 0, Yes = 1
Technologies used	
x61	Maternal and child care = 0, Public health = 1
x62	Maternal and child care = 0, Traditional Chinese medicine = 1
x63	Maternal and child care = 0, Family planning = 1
x64	Maternal and child care = 0, Chronic conditions = 1

## Results

### Characteristics of respondents

Health worker participants were on average 37 years old, and more than half (53.5 %) were women. Those in their thirties accounted for 48 % of the respondents. About 43 % held a junior professional title and 54.0 % had a university degree. The top five areas of services provided by the respondents were gynecology and obstetrics, pediatrics, TCM, internal medicine, and surgery. As many as 39 % of respondents had less than 10 years of work experience and 60 % earned less than 3000 Yuan a month.

The average age of patient participants was 39 years, and once again females in the majority (61 %). Most respondents (67.8 %) had only completed a junior high level of education (Year 10) and 61 % were covered by the New Rural Cooperative Medical Scheme (NCMS), a government subsidized voluntary social health insurance scheme [16]. NCMS enrollees are eligible to have their AHT service expenses reimbursed. About 44 % of patient participants had an annual household income of less than 30000 Yuan.

### Knowledge, attitudes and behaviors of health workers towards AHTs

All of the health worker participants were involved in the delivery of at least one AHT and had received training in relation to those technologies. The majority of respondents (74.1 %) were satisfied with the promotion of AHTs, with 11.2 % reporting feelings of being “very satisfied”.

Significant differences in the aggregate scores in relation to the use of AHTs appeared across areas of services. The respondents delivering public health services gave the highest scores (*p* < 0.01) to AHTs (4.42 ± 0.57), followed by those engaged in management of chronic conditions (4.41 ± 0.57) and traditional Chinese medicine (4.29 ± 0.55). Male respondents and those who held a senior professional title gave a higher score (*p* < 0.01) to AHTs than their colleagues (Table 3).

**Table 3:** Aggregate scores given by health workers in relation to the use of AHTs

Characteristics of respondents	Aggregate score ($\overline{X}\pm S$)	U or *χ* ^*2*^	p*
Gender			
Male	4.35 ± 0.57	74101.0	0.002
Female	4.19 ± 0.66		
Age (year)			
≤29	4.29 ± 0.59	0.286	0.963
30-39	4.28 ± 0.61		
40-49	4.25 ± 0.59		
≥50	4.22 ± 0.82		
Level of Education			
≤Junior high school	4.10 ± 0.76	4.884	0.181
Senior high (vocational) school	4.30 ± 0.57		
Associate degree	4.22 ± 0.61		
≥Bachelor degree	4.29 ± 0.65		
Monthly Income (Yuan)			
<1000	4.41 ± 0.51	6.326	0.097
1000-2999	4.21 ± 0.68		
3000-4999	4.34 ± 0.55		
≥5000	4.26 ± 0.71		
Professional Title			
No	4.26 ± 0.46	13.262	0.004
Junior	4.26 ± 0.61		
Middle	4.22 ± 0.69		
Senior	4.47 ± 0.54		
Work Experience (year)			
≤9	4.31 ± 0.62	2.465	0.482
10-19	4.27 ± 0.58		
20-29	4.26 ± 0.64		
≥30	4.13 ± 0.82		
Areas of Services^#^			
(1) Public health	4.42 ± 0.57	17.169	0.002
(2) Maternal and child care	4.16 ± 0.75		
(3) Traditional Chinese medicine	4.29 ± 0.55		
(4) Family planning	4.23 ± 0.41		
(5) Chronic condition	4.41 ± 0.57		

*Mann–Whitney U or Kruskal-Wallis H test

^#^Nemenyi Post Hoc tests found statistical significance (*p* < 0.05): (1) vs (2); (1) vs (4); (2) vs (5); (4) vs (5)

In general, the majority of health workers expressed strong willingness to adopt AHTs. As many as 90 % of these respondents believed that AHTs were meaningful for rural patients and therefore were willing to adopt and use those technologies. Despite this, differences in attitudes towards specific technologies were evident: 69 % of health workers believed that the technologies encouraged by the government were mature and a quarter acknowledged difficulties in using those technologies. Only 23 % of respondents felt that the AHTs they were delivering should be scaled up; 73 % recommended limited use of the AHTs they were delivering; while 2 % objected to the promotion of the AHTs they were delivering. TCM workers obtained the highest score in acceptance of their applied AHTs compared with family planning workers, who rated the lowest score on their applied AHTs (*p* < 0.001, Table 4).

**Table 4:** Dimensional scores ($\overline{X}\pm S$) given by health workers in relation to the use of AHTs

Dimension	Public health	Maternal and child health	Traditional Chinese medicine	Family planning	Chronic conditions	*χ* ^*2*^	p*
Knowledge about AHTs	3.81 ± 0.48	3.69 ± 0.57	3.85 ± 0.46	3.43 ± 0.47	3.80 ± 0.48	42.648	0.000
Perceived impact of AHTs on patients	3.97 ± 0.56	3.67 ± 0.61	3.88 ± 0.53	3.90 ± 0.51	3.95 ± 0.45	35.938	0.000
Perceived impact of AHTs on providers	3.53 ± 0.43	3.46 ± 0.48	3.58 ± 0.45	3.33 ± 0.39	3.62 ± 0.40	35.785	0.000
Satisfaction on training & management	4.10 ± 0.45	3.82 ± 0.56	4.03 ± 0.47	3.92 ± 0.46	4.02 ± 0.46	32.602	0.000

*Kruskal-Wallis H test

Nevertheless, health workers gave a high rating on the impact of AHTs on patients. More than 75 % respondents acknowledged the acceptance of those technologies from patients; 71 % believed that AHTs reduced the financial burden of patients; and 78 % acknowledged patient satisfaction with the AHTs they received. Public health workers gave the highest rating on this dimension, followed by those engaged in the management of chronic conditions. Once again, the lowest rating came from maternal and child health care workers (*p* < 0.001, Table 4).

Health workers gave a positive yet relatively low rating on the impact of AHTs with respect to providers, perhaps because the impact of AHTs on income fell short of expectation. Fewer than 25 % of respondents believed that their personal income increased, while only a slightly higher percentage (38 %) believed their departmental income increased because of the use of AHTs. Overall, those who engaged in the management of chronic conditions and public health services favorably rated the impact of AHTs benefitting providers financially compared with their colleagues (*p* < 0.001, Table 4).

Health workers were also highly satisfied with AHT training and managerial arrangements: 84 % of respondents reported their managers held AHTs high in their agenda, and 79 % were satisfied with the management procedures. However, fewer than 60 % of respondents were happy with the input and management of funding and 49 % did not believe adequate assessment and incentive measures had been established. More than 72 % of respondents believed that lack of assessment and incentives would jeopardize the promotion of AHTs. Overall, public health workers had a higher degree of satisfaction on training and management arrangements than their colleagues (*p* < 0.001, Table 4).

The regression analysis proved that health workers’ rating on AHTs was associated with areas of services, working experience, and financial incentives (*p* < 0.05). Those who engaged in services other than maternal and child health care and those who were less experienced, in a higher pay scale or were financially encouraged to deliver AHTs rated them higher compared with their colleagues (Table 5).

**Table 5:** Factors associated with the overall rating of health workers on AHTs: results of regression analysis

	*B*	*(95 % CI)*	*Beta*	*t*	*p*
Constant	3.464	(3.131, 3.796)		20.452	0.000
Income	0.061	(0.011, 0.112)	0.086	2.382	0.017
Work experience	−0.095	(−0.148, −0.042)	−0.205	−3.508	0.000
Areas of services					
Public health	0.209	(0.099, 0.318)	0.137	3.749	0.000
Traditional Chinese medicine	0.135	(0.055, 0.214)	0.129	3.330	0.001
Family planning	0.103	(0.001, 0.204)	0.074	1.981	0.048
Management of chronic conditions	0.188	(0.102, 0.274)	0.165	4.293	0.000
Incentives	0.148	(0.088, 0.209)	0.167	4.796	0.000

### Knowledge, attitudes and behaviors of patients towards AHTs

Overall, patients were satisfied with AHTs, with 71.6 % feeling “very satisfied” or “satisfied”, 24.2 % feeling “acceptable” and 1.6 % feeling “dissatisfied”. Significant differences existed in the degree of satisfaction of patients towards different areas of services (*χ*^2^ = 37.548, *p* < 0.001). About 83 % of patients were satisfied or very satisfied with TCM, compared with 80 % with management of chronic conditions, 67 % with family planning, 64 % with public health services, and 59 % with maternal and child health care.

In terms of technical effectiveness, higher degrees of patient satisfaction were found in management of chronic conditions and TCM. Patients were least satisfied with maternal and child health care (*p* < 0.001). The degree of patient satisfaction on technical effectiveness was directly proportional with household income and insurance coverage levels (*p* < 0.01, Table 6).

**Table 6:** Patient satisfaction on technical effectiveness of AHTs

	$\overline{X}\pm S$	(U) *χ* ^*2*^	p*
Gender			
Male	4.04 ± 0.66	56390.500	0.756
Female	4.06 ± 0.65		
Age (year)			
<20	4.02 ± 0.68	2.981	0.395
20-39	4.00 ± 0.71		
40-59	4.13 ± 0.59		
≥60	4.05 ± 0.62		
Level of education			
≤Junior high school	4.03 ± 0.65	5.646	0.130
Senior high (vocational) school	4.02 ± 0.68		
Associate degree	4.16 ± 0.69		
≥Bachelor degree	4.23 ± 0.60		
Annual household income (yuan)			
<10000	3.98 ± 0.62	14.401	0.002
10000-29999	3.99 ± 0.67		
30000-49999	4.02 ± 0.64		
≥50000	4.20 ± 0.67		
Area of services^a^			
(1) Public health	4.02 ± 0.75	31.452	0.000
(2) Maternal and child care	3.84 ± 0.69		
(3) Traditional Chinese medicine	4.16 ± 0.58		
(4) Family planning	4.11 ± 0.61		
(5) Management of chronic conditions	4.17 ± 0.63		
Insurance coverage			
No	3.82 ± 0.64	13635.500	0.003
Yes	4.07 ± 0.65		

*Mann–Whitney U or Kruskal-Wallis H test

^a^Nemenyi Post Hoc tests found statistical significance (*p* < 0.05): (2) vs (3); (2) vs (4); (2) vs (5)

About half of patients rated the pricing of AHTs as “generally acceptable”. Public health services, TCM and family planning services were more likely to be rated as low in prices compared with maternal and child health care and management of chronic conditions (*p* < 0.001, Table 7). The patient rating on pricing of AHTs was associated with household incomes (*p* <0.01), but not with insurance compensation (*p* > 0.5).

**Table 7:** Patient rating on the level of price of AHTs, n (%)

	Very high	High	Average	Low	Very low	*χ* ^*2*^	*p*
Gender							
Male	13(4.8 %)	34(12.5 %)	131(48.3 %)	66(24.4 %)	27(10.0 %)	7.336	0.119
Female	16(3.8 %)	44(10.4 %)	213(50.5 %)	82(19.4 %)	67(15.9 %)		
Age (year)							
<20	6(5.4 %)	9(8.1 %)	56(50.5 %)	21(18.9 %)	19(17.1 %)	12.528	0.404
20-39	9(3.5 %)	32(12.3 %)	138(53.1 %)	46(17.7 %)	35(13.5 %)		
40-59	7(3.8 %)	22(11.8 %)	94(50.5 %)	44(23.7 %)	19(10.2 %)		
≥60	7(5.1 %)	15(11.0 %)	56(41.2 %)	37(27.2 %)	21(15.4 %)		
Level of education							
≤Junior high school	19(4.0 %)	48(10.2 %)	225(47.9 %)	106(22.6 %)	72(15.3 %)	14.941	0.245
Senior high school	2(1.6 %)	14(11.5 %)	66(54.1 %)	26(21.3 %)	14(11.5 %)		
Associate degree	5(7.0 %)	11(15.5 %)	38(53.5 %)	12(16.9 %)	5(7.0 %)		
≥Bachelor degree	3(10.0 %)	5(16.7 %)	15(50.0 %)	4(13.3 %)	3(10.0 %)		
Annual household income (yuan)							
<10000	9(5.9 %)	25(16.4 %)	72(47.4 %)	35(23.0 %)	11(7.2 %)	29.349	0.003
10000-29999	6(4.0 %)	20(13.2 %)	67(44.4 %)	26(17.2 %)	32(21.2 %)		
30000-49999	3(1.4 %)	20(9.2 %)	118(54.4 %)	46(21.2 %)	30(13.8 %)		
≥50000	11(6.4 %)	13(7.5 %)	87(50.3 %)	41(23.7 %)	21(12.1 %)		
Area of services^#^							
Public health	2(5.0 %)	3(7.5 %)	8(20.0 %)	5(12.5 %)	22(55.0 %)	192.54	0.000
Maternal and child care	6(2.8 %)	35(16.4 %)	139(65.3 %)	32(15.0 %)	1(0.5 %)		
Traditional Chinese medicine	11(5.3 %)	10(4.8 %)	97(46.9 %)	70(33.8 %)	19(9.2 %)		
Family planning	5(5.5 %)	2(2.2 %)	37(40.7 %)	13(14.3 %)	34(37.4 %)		
Management of chronic conditions	5(3.5 %)	28(19.7 %)	63(44.4 %)	28(19.7 %)	18(12.7 %)		
Insurance coverage							
No	2(3.6 %)	12(21.4 %)	21(37.5 %)	14(25.0 %)	7(12.5 %)	7.895	0.096
Yes	27(4.2 %)	66(10.4 %)	323(50.7 %)	134(21.0 %)	87(13.7 %)		

^#^Paired comparisons:

“Public health” vs “Maternal and child care” (*x* ^*2*^=41.659, *P* < 0.001)

“Public health” vs “Traditional Chinese medicine” (*x* ^*2*^=11.908, *P* = 0.003)

“Public health” vs “Management of chronic conditions” (*x* ^*2*^=22.907, *P* < 0.001)

“Maternal and child care” vs “Traditional Chinese medicine” (*x* ^*2*^=27.975, *P* < 0.001)

“Maternal and child care” vs “Family planning” (*x* ^*2*^=45.106, *P* < 0.001)

“Traditional Chinese medicine” vs “Family planning” (*x* ^*2*^=6.672, *P* = 0.036)

“Family planning” vs “Management of chronic conditions” (*x* ^*2*^=19.018, *P* < 0.001)

The regression analysis proved that patient rating for AHTs was associated with the variables of service area, age, and household income (*p* < 0.05). A higher degree of patient satisfaction was found in those who received services in relation to management of chronic conditions, TCM, family planning and those who were younger and had a higher household income compared with others (Table 8).

**Table 8:** Factors associated with the overall rating of patients on AHTs: results of regression analysis

	*B*	*(95 % CI)*	*Beta*	*t*	*P*
Constant	3.749	(3.487, 4.011)		28.076	<0.001
Age	−0.003	(−0.006, 0.001)	−0.098	−2.091	0.037
Household income	0.054	(0.008, 0.101)	0.090	2.286	0.023
Area of services					
Traditional Chinese medicine	0.376	(0.236, 0.515)	0.262	5.274	0.000
Family planning	0.306	(0.142, 0.469)	0.158	3.672	0.000
Management of chronic conditions	0.463	(0.291, 0.635)	0.285	5.292	0.000

## Discussion

AHTs have been endorsed in many countries, such as Argentina, Cuba, Saudi Arabia and Thailand. They have been proved to be cost-effective through randomized controlled trials [3, 17].

This study demonstrated that AHTs have been widely accepted by health workers in China. More than 90 % of health workers agreed that AHTs could benefit their rural patients; however, satisfaction levels of both health workers and patients fell short of expectation. Only about 72 % of health workers and patients were satisfied with their AHTs. This finding is similar to the results of studies undertaken elsewhere [18, 19]. Despite differences in demographic characteristics and disease profiles of participants, Peng [18] and Sun [19] found similar levels of patient satisfaction on AHTs.

In China, AHTs are mainly defined by the Ministry of Health and regional (provincial and municipal) health authorities. Local governments (counties and districts) who fund primary care services have limited authority over the determination of AHTs although some technologies may not be appropriate to their local contexts [20], which may explain the low levels of AHT satisfaction amongst health workers. It is imperative to match AHTs with local needs and capacities: AHTs can only become effective when local health workers deliver them in an appropriate and effective way.

Participants (both health workers and consumers) of this study gave a low score to AHTs associated with maternal and child care; regarding them as costly and having limited patient benefit. This is perhaps because the government-defined maternal and child care AHTs are somewhat limited and not specifically adapted to the needs of rural populations [21].

Although AHTs associated with the management of chronic conditions were perceived by patients as costly, their technical effectiveness was recognized by both health workers and patients. Drug therapy is an essential AHT component for managing chronic conditions. Patients usually need to take medicines for the rest of their lives, incurring expensive medical bills [22].

Interestingly, AHTs associated with TCM was rated relatively high by both health workers and patients. Although many of those traditional technologies have not been fully supported by scientific evidence, they are usually cheap and highly favored by patients [23]. In some medical institutions, TCM are provided free of charges [24].

Public health services were regarded as highly effective by health workers, but conversely low by patients. Compared with other AHTs, public health services may bring benefits to populations without necessarily being seen as beneficial to individuals [25].

This study revealed that the lack of financial incentives may jeopardize the use of AHTs. Although financial incentives were overwhelmingly (72 %) regarded as important for encouraging health workers to adopt AHTs, fewer than half of the health workers believed their efforts were adequately compensated. Because of the low fee levels attached to AHTs, they may result in a financial disadvantage to health workers. Some researchers argue that AHTs should be treated as a public good [26], with funding support from governments. In Zhejiang province, the promotion of AHTs is included in the performance assessment of local health administration in some counties [27, 28].

## Conclusions

Overall, both health workers and patients were willing to endorse AHTs. AHTs were believed to be able to bring health and financial benefits to patients. However, doubts exist among health workers regarding the maturity, evidence and technical applicability of the selected AHTs. Affordability of the AHTs is a serious concern for patients. They tended to express a higher level of dissatisfaction towards services that are likely to incur higher levels of expense, such as maternal and child health care and management of chronic conditions. Public health services, despite its relatively lower costs, were also unfavorably rated in patient satisfaction.

Perspectives from health workers and patients are equally important in determining AHTs. Although health workers play a critical role in adopting and implementing AHTs, patients are demanding greater participation in decision making. Local needs and applicability should be taken into consideration when determining the introduction of AHTs. It would also seem that consumer health literacy needs to be improved, in particular in relation to public health and preventive services.

This study was undertaken in one province (Zhejiang): generalization of the results to other regions of China should be treated with caution.
